# Pictorial Essay on Ultrasound and Magnetic Resonance Imaging of Paraspinal Muscles for Myofascial Pain Syndrome

**DOI:** 10.3390/life14040499

**Published:** 2024-04-12

**Authors:** Chen-Yu Hung, Bow Wang, Hsiang-Chi Chang, Wei-Ting Wu, Ping-Tang Liu, Ke-Vin Chang, Daniel Chiung-Jui Su, Kamal Mezian, Vincenzo Ricci, Levent Özçakar

**Affiliations:** 1 Department of Physical Medicine and Rehabilitation, National Taiwan University Hospital, Bei-Hu Branch, Taipei 10845, Taiwan; chenyu810@gmail.com (C.-Y.H.); wwtaustin@yahoo.com.tw (W.-T.W.); 2 Department of Medical Imaging, National Cheng Kung University Hospital, College of Medicine, National Cheng Kung University, Tainan 704302, Taiwan; wangbow1227@gmail.com; 3 Department of Physical Medicine and Rehabilitation, Taipei Hospital, Ministry of Health and Welfare, Taipei 24213, Taiwan; sss19801041@gmail.com; 4 Institute of Public Health, National Yang Ming Chiao Tung University, Taipei 11221, Taiwan; 5 Department of Physical Medicine and Rehabilitation, College of Medicine, National Taiwan University, Taipei 10048, Taiwan; 6 Jolly Clinics, Kaohsiung 80232, Taiwan; morgan0831mm@yahoo.com.tw; 7 Center for Regional Anesthesia and Pain Medicine, Wang-Fang Hospital, Taipei Medical University, Taipei 11600, Taiwan; 8 Department of Physical Medicine and Rehabilitation, Chi-Mei Medical Center, Tainan 71004, Taiwan; lovedaniel@gmail.com; 9 Department of Rehabilitation Medicine, First Faculty of Medicine and General University Hospital in Prague, Charles University, 12800 Prague, Czech Republic; kamal.mezian@gmail.com; 10 Physical and Rehabilitation Medicine Unit, Department of Biomedical and Neuromotor Science, Istituto di Ricovero e Cura a Carattere Scientifico Rizzoli Orthopedic Institute, 40136 Bologna, Italy; vincenzo.ricci58@gmail.com; 11 Department of Physical and Rehabilitation Medicine, Hacettepe University Medical School, Ankara 06100, Turkey; lozcakar@yahoo.com

**Keywords:** pain, sonography, neck, trunk, lumbar

## Abstract

The paraspinal muscles of the cervical, thoracic, and lumbar spine are important pain generators because muscle strains or myofascial pain syndrome caused by trigger points are common during clinical practice. Ultrasonography is the most convenient imaging tool for evaluating these muscles due to its advantages, such as providing good delineation of soft tissues, easy accessibility, and zero radiation. Additionally, ultrasound can serve as a useful guiding tool for paraspinal muscle intervention to prevent inadvertent injuries to vital axial neurovascular structures. This pictorial essay presents ultrasound scanning protocols for the paraspinal and other associated muscles as well as a discussion of their clinical relevance. Axial magnetic resonance imaging has also been used to elucidate reciprocal anatomy. In conclusion, ultrasound imaging proves to be a valuable tool that facilitates the differentiation of individual paraspinal muscles. This capability significantly enhances the precision of interventions designed to address myofascial pain syndrome.

## 1. Introduction

The paraspinal muscles can generally be divided into three groups. The superficial extrinsic back muscles assist in the movement of the upper limbs, including the trapezius, levator scapulae, latissimus dorsi, rhomboid major, and rhomboid minor. The intermediate extrinsic back muscles facilitate respiration, including the serratus posterior superior and serratus posterior inferior. The intrinsic back muscles are responsible for the motion of the axial skeleton and can be further divided into three layers. The superficial layer consists of the splenius muscles located in the lateral and posterior neck region. The intermediate layer, also known as the erector spinae, spans throughout the lumbar, thoracic, and cervical regions and contains the iliocostalis, longissimus, and spinalis. The deep layer, also categorized as the transversospinalis, is located between the transverse processes and spinous processes of the vertebrae and comprises the semispinalis, multifidus, and rotatores [1]. This article aims to address the extrinsic/intrinsic paraspinal and associated muscles using ultrasound imaging, which are also correlated with magnetic resonance imaging. The spinalis of the erector spinae and the rotatores of the transversospinalis are not discussed in this article as they are difficult to differentiate from adjacent muscles on ultrasound imaging [2].

Concerning the utilization of imaging modalities for assessing muscles with myofascial pain, Mazza et al.’s systematic review [3] identified sonoelastography as the most effective ultrasound-based technique for evaluating trigger points or taut bands in patients with myofascial pain syndrome. Alternative ultrasound methods include enhancing simple B-mode ultrasound images through texture analysis and entropy filtering, assessing the echogenicity of muscles with trigger points, or employing Doppler ultrasound imaging to evaluate blood flow in affected muscles. Some studies incorporated magnetic resonance imaging techniques, such as magnetic resonance elastography and T2 mapping, demonstrating the capability of visualizing myofascial trigger points.

This pictorial essay’s principal objective is not an exhaustive examination of the various literature; rather, it systematically presents ultrasound scanning methods derived from existing research, which is important for the guided injection of myofascial pain. Our focus is not to delve into a discussion about the correlation between ultrasound and magnetic resonance imaging. Instead, acknowledging the widespread use of axial magnetic resonance imaging in illustrating the topography of paraspinal muscles, we utilize magnetic resonance imaging as a reference for identifying the paraspinal muscles in our ultrasound images.

To create the pictorial essay, the literature search primarily utilized PubMed to identify pertinent articles, without any language restrictions. The search spanned from its inception to March 2024, encompassing various article types as long as they were relevant to the ultrasound or magnetic resonance imaging of paraspinal muscles and the treatment of myofascial pain syndrome. The keywords employed included “ultrasound”, “sonography”, “magnetic resonance imaging”, “paraspinal muscle”, “trigger point”, and “myofascial pain”.

## 2. Superficial Extrinsic Muscles

### 2.1. Trapezius and Latissimus dorsi

#### 2.1.1. Anatomy

**Figure 1:**
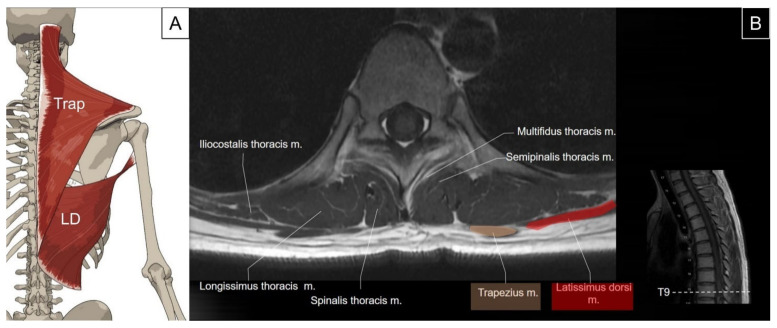
Illustration (**A**) and axial view of magnetic resonance imaging (**B**) for the trapezius (Trap) and latissimus dorsi (LD) muscle.

The trapezius is the most superficial muscle overlying the posterior neck and upper back. It has a flat and triangular shape, while the paired muscles resemble a trapezoid, from which the name is derived. The muscle originates from the medial third of the superior nuchal line, external occipital protuberance, ligamentum nuchae, and the apices of the spinous processes and supraspinous ligaments (from C7 to T12). It inserts bilaterally on the posterior border of the lateral one-third of the clavicle, the acromion process, and the spine of the scapula. The main function of the trapezius is to stabilize and move the scapula [2]. In addition, the upper fibers of the trapezius serve as neck extensors [4]. The latissimus dorsi muscle is also flat and triangular in shape, originating from the spinous processes of the lower six thoracic vertebrae, thoracolumbar fascia, supraspinous ligaments, and the posterior part of the iliac crest [5]. It sweeps over the lower thorax and lumbar regions, having attachments on the lower three to four ribs where it interdigitates with the external oblique muscle and inferior angle of the scapula. It then extends toward the axilla, winding around the anterior aspect of the teres major muscle to insert on the floor of the intertubercular sulcus. Contraction of the latissimus dorsi leads to extension, adduction, and internal rotation of the humerus (Figure 1) [2].

#### 2.1.2. Sonographic Scanning

**Figure 2:**
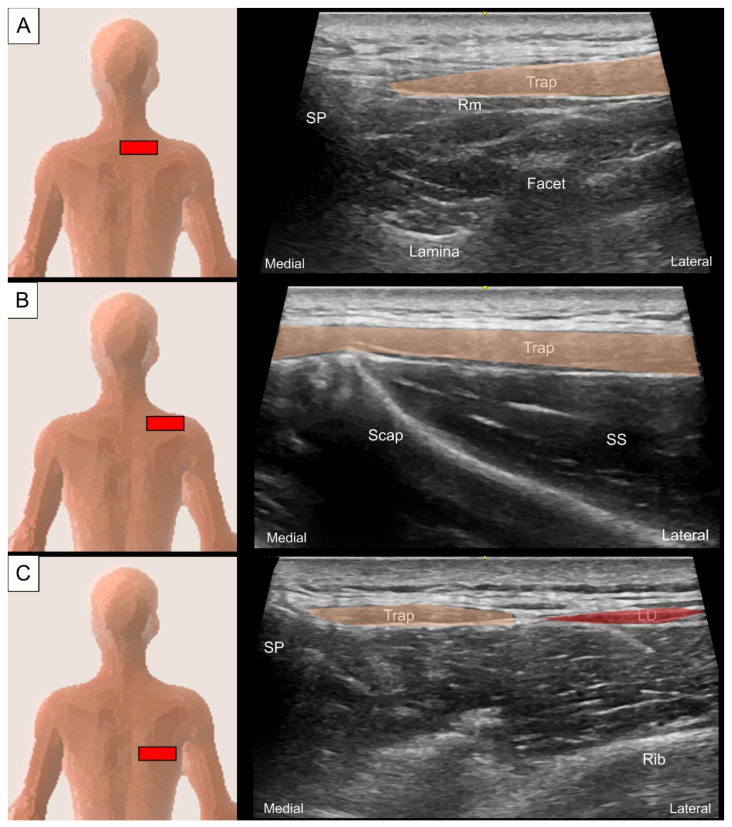
Ultrasound imaging of the upper trapezius (Trap, skin color shade) muscle at around the first thoracic vertebral level near the (**A**) midline and (**B**) on top of the supraspinatus (SS) muscle; ultrasound imaging of the middle trapezius and latissimus dorsi (LD, red shade) muscle at the eighth thoracic level (**C**). Rm, rhomboid minor; SP, spinous process; Scap, scapula.

All sonographic images in this and the following sessions were recorded using a 4–15 MHz linear and a 1–6 MHz curved transducer (LOGIQ S8, GE Healthcare, Chicago, IL, USA) with the subject in a prone or side-lying posture. To scan the trapezius, place the transducer transversely at the suboccipital region, adjacent to the spinous process. At this level, the trapezius appears as a very thin muscle superficial to the semispinalis capitis on the medial aspect. When the transducer is slid caudally towards the upper thoracic region, the muscle appears thicker and wider with its midline attaching to the thoracic spinous processes (Figure 2A). On the lateral aspect, it extends to the acromion process, covering the supraspinatus muscle (Figure 2B). Sliding the transducer further caudally towards the lower thoracic levels reveals the latissimus dorsi, which emerges as a thin muscle belly deep and lateral to the lower trapezius (Figure 2C). Conversely, the latissimus dorsi becomes the most superficial back muscle at the thoracolumbar junction.

#### 2.1.3. Clinical Relevance

Myofascial pain syndrome related to trigger points in the trapezius muscle is a common occurrence in clinical practice. The resulting pain can radiate to the head, neck, shoulder, suprascapular, or interscapular regions. Ultrasound-guided injections into the trapezius can help avoid inadvertent injection into neurovascular structures, such as the superficial and deep branches of the transverse cervical artery and the spinal accessory nerve [6,7]. The latissimus dorsi injury could occur in isolation or in combination with the pectoralis major, teres major, or rotator cuff muscles. Avulsion injuries at the humeral attachment or myotendinous junction are common injuries reported [8].

### 2.2. Rhomboids

#### 2.2.1. Anatomy

**Figure 3:**
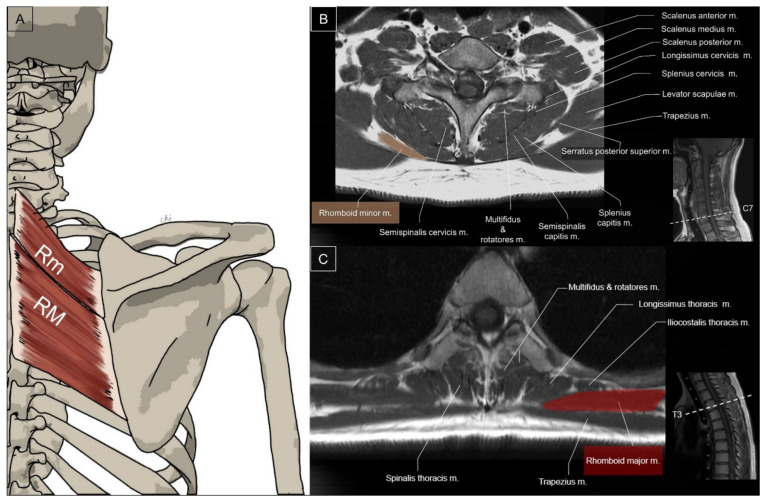
Illustration (**A**) and axial view of magnetic resonance imaging for the rhomboid minor (Rm) (**B**) and rhomboid major (RM) muscles (**C**).

The rhomboid minor is a small, cylindrical muscle that originates from the lower nuchal ligament and the spinous processes of the C7 and T1 vertebrae [9]. It inserts on a small area of the medial border of the scapula at the level of the scapular spine. On the other hand, the rhomboid major muscle has a quadrilateral shape and originates from the spinous processes of the T2 to T5 vertebrae [10]. Its insertion is on the medial border of the scapula just inferior to the rhomboid minor. The rhomboid major and minor have parallel fibers and may be fused together to form a single muscle. The rhomboid muscle group retracts the medial border of the scapula toward the superior and medial aspect to facilitate shoulder retraction (Figure 3) [11].

#### 2.2.2. Sonographic Scanning

**Figure 4:**
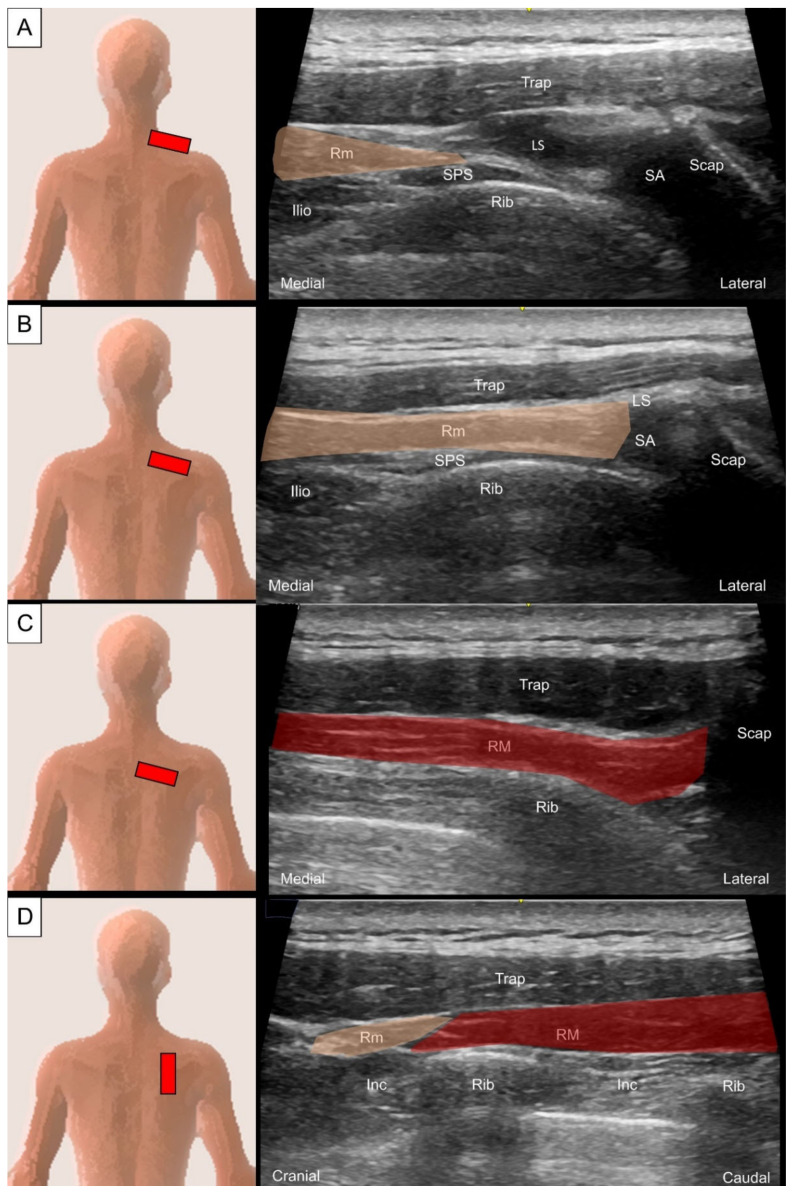
Ultrasound imaging of the rhomboid minor (Rm, skin color shade) at the (**A**) more craniomedial and (**B**) more caudolateral aspect, and (**C**) rhomboid major (RM, red color shade) in the horizontal oblique plane; ultrasound imagining of the rhomboid muscle group (**D**) in the sagittal plane. Tra, trapezius; SPS, serratus posterior superior; LS, levator scapulae; SA, serratus anterior; Scap, scapula; Inc, intercostal muscle.

The transducer should be placed adjacent to the midline at the cervicothoracic junction on the examined side and oriented with a lateral-inferior direction towards the scapula spine. The rhomboid minor can be visualized as a quadrangular muscle located underneath the trapezius with a thin fascial attachment on the spinous process (Figure 4A). Differentiating the rhomboid minor from the levator scapulae near their insertion on the medial scapula border could be challenging. The rhomboid minor is situated more medially than the levator scapulae. Additionally, while scanning more caudally, the levator scapulae continuously becomes smaller and eventually becomes a small hyperechoic tendinous slip (Figure 4B) [12].

When the transducer is moved caudally from its position over the rhomboid minor, the same orientation can be used to visualize the rhomboid major (Figure 4C). Rotating the transducer 90 degrees allows for visualization of the short axis of the rhomboid muscle group, which shows a thin inter-muscular fascial separation (Figure 4D).

#### 2.2.3. Clinical Relevance

The rhomboid minor and major are important stabilizers of the scapula. Damage to the dorsal scapular nerve can cause rhomboid muscle palsy and subsequent winging of the scapula. When performing interventions such as trigger point injections on the rhomboid muscle, clinicians should be aware of the potential risk of inadvertent dorsal scapular nerve injury [13,14].

### 2.3. Levator Scapulae

#### 2.3.1. Anatomy

**Figure 5:**
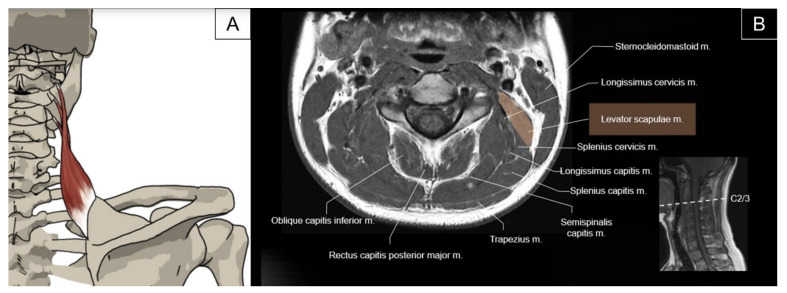
Illustration (**A**) and axial view of magnetic resonance imaging (**B**) for the levator scapulae muscle.

The levator scapulae muscle originates from the posterior tubercle of the transverse process of the C1 to C4 vertebrae and inserts on the superior medial border of the scapula. The primary action of the levator scapulae is to elevate the scapula [15]. Together with other posterior axial-appendicular muscles, the levator scapulae can depress the scapula and tilt the glenoid cavity inferiorly by rotating the scapula downward. Contraction of the levator scapulae also contributes to extending and laterally flexing the neck (Figure 5) [2].

#### 2.3.2. Sonographic Scanning

**Figure 6:**
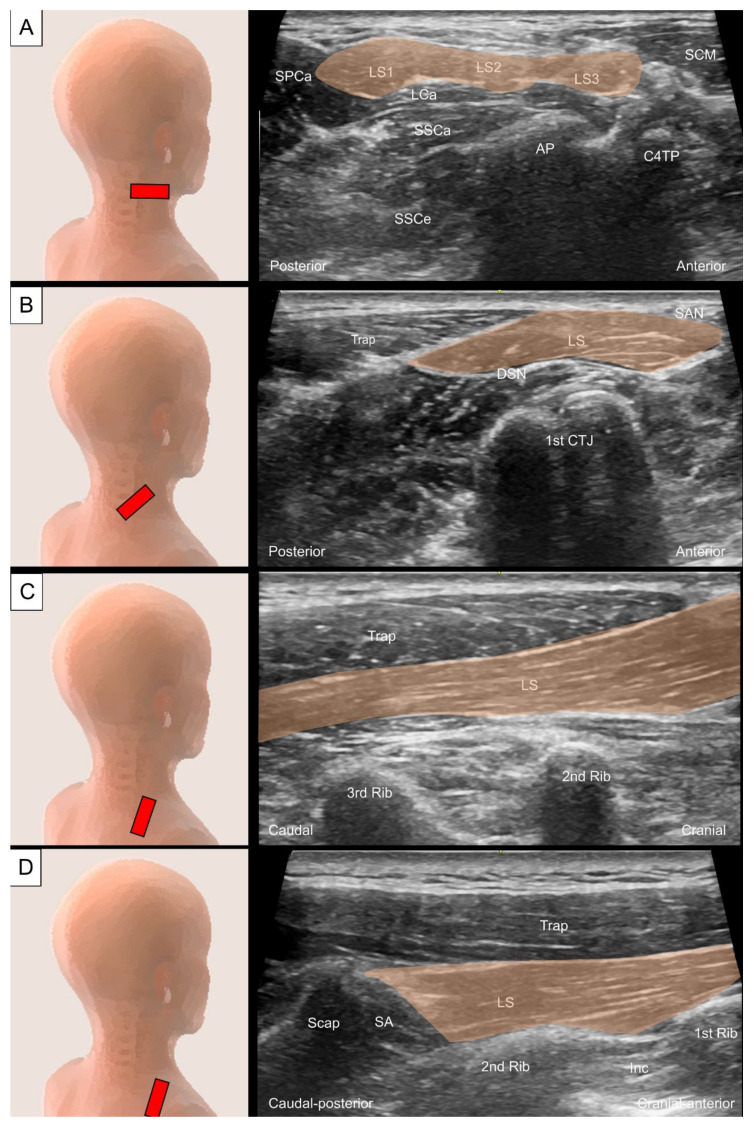
Ultrasound imaging of (**A**) the levator scapulae (LS, skin color shade) emerging from the C1 to C4 transverse processes, and (**B**) the spinal accessory nerve (SAN) and dorsal scapular nerve (DSN) besides the levator scapulae. (**C**) Ultrasound imaging for the long-axis view of the levator scapulae and (**D**)its tendinous insertion. Numbers 1, 2, 3 indicate different bundles of the levator scapulae. SCM, sternocleidomastoid; SPCa, splenius capitis; LCa, longissimus capitis; SSCa, semispinalis capitis; SSCe, semispinalis cervicis; AP, articular process; C4TP, transverse process of the fourth cervical vertebrae; Trap, trapezius; CTJ, costotransverse joint; SA, serratus anterior; Scap, scapula.

To scan the axial view of the proximal levator scapulae, the transducer is placed transversely on the lateral neck at the level of the transverse process of C1, just inferior to the mastoid process. The transducer is then slid caudally to visualize the individual muscle belly emerging from the C1 to C4 transverse processes (Figure 6A). The sternocleidomastoid (SCM) muscle is seen superficial to the proximal levator scapulae. The four muscle bellies form a common muscle coursing underneath the trapezius muscle at the junction of the neck and shoulder. The spinal accessory nerve is usually seen as a hyperechoic oval structure between the trapezius and the levator scapulae (Figure 6B). The dorsal scapular nerve can be seen coursing within the fascial plane deep to the levator scapulae [16]. The long-axis view of the levator scapulae can be obtained by placing one end of the transducer towards the superior medial border of the scapula and the other end towards the transverse process of C4 or above (Figure 6C). To better evaluate distal tendinous pathologies, such as enthesopathy, the transducer could be directed to the inner aspect of the scapula (Figure 6D).

#### 2.3.3. Clinical Relevance

The enthesopathy of the levator scapulae commonly causes medial scapular pain [17]. Intervention to the levator scapulae can be performed under ultrasound guidance to avoid inadvertent injury to the spinal accessory nerve and the dorsal scapular nerve [18].

## 3. Intermediate Extrinsic Muscles

### 3.1. Serratus Posterior Superior/Inferior

#### 3.1.1. Anatomy

**Figure 7:**
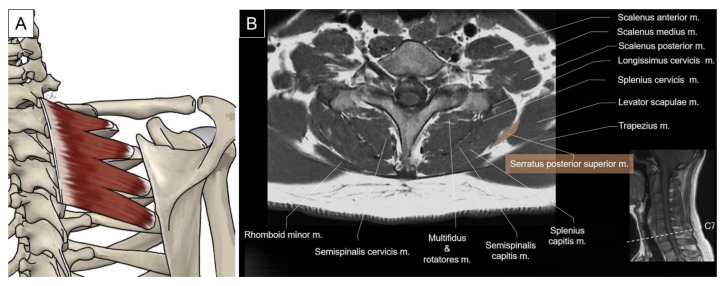
Illustration (**A**) and axial view of magnetic resonance imaging (**B**) for the serratus posterior superior muscle.

The serratus posterior superior is a thin quadrilateral muscle that lies deep to the rhomboid muscle group. It originates as a thin aponeurosis from the inferior part of the nuchal ligament, the spinous processes of the C7 to T3 vertebrae, and the supraspinous ligaments. It courses inferiorly and laterally with four digitations attaching onto the superior margin of the second to fifth ribs. The serratus posterior superior helps with deep inspiration by elevating the attached ribs (Figure 7) [2].

**Figure 8:**
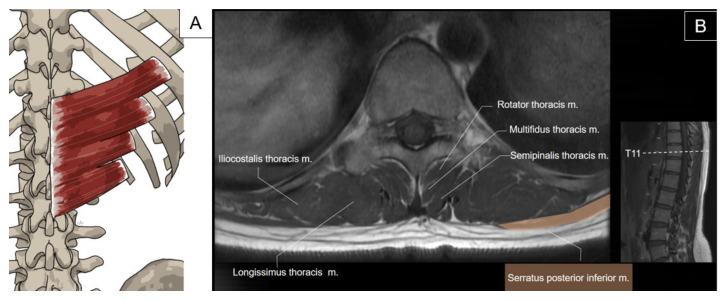
Illustration (**A**) and axial view of magnetic resonance imaging (**B**) for the serratus posterior inferior muscle.

The serratus posterior inferior is a thin, irregularly quadrilateral muscle located on the posterior trunk at the thoracolumbar junction. Its origin includes the spinous processes of the lower two thoracic and upper two to three lumbar vertebrae, as well as the supraspinous ligaments, which blend with the thoracolumbar fascia. It courses superolaterally to insert on the inferior borders of the lower four ribs lateral to the rib angles. This muscle acts as a rib depressor, aiding in expiration (Figure 8) [2,19].

#### 3.1.2. Sonographic Scanning

**Figure 9:**
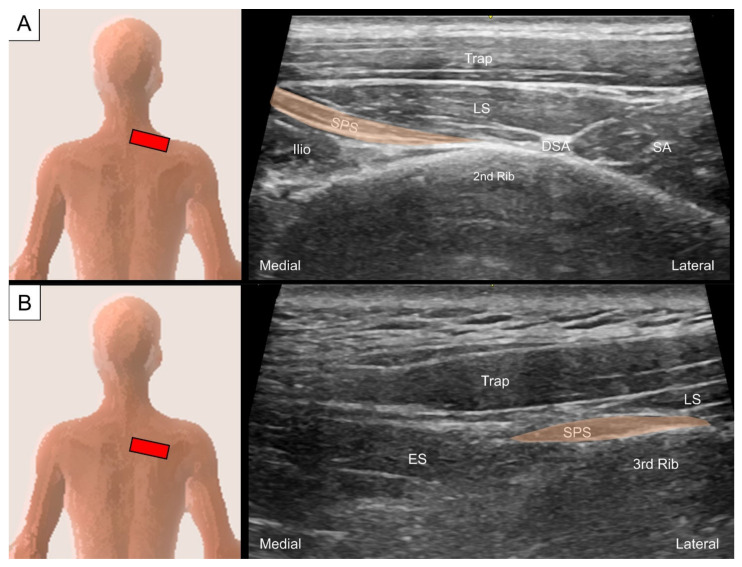
Ultrasound imaging of the serratus posterior superior (SPS) attaching to the (**A**) second and (**B**) third ribs. Trap, trapezius; LS, levator scapulae; SA, serratus anterior; DSA, dorsal scapular artery; ilio, iliocostalis; ES, erector spinae.

To locate the serratus posterior superior, the transducer is placed at the cervical–thoracic junction just lateral to the C7 spinous process. The transducer is tilted toward the caudal direction with the footprint perpendicular to the skin to visualize the uppermost digitation of the serratus posterior superior as a thin muscle belly underneath the levator scapulae. This muscle courses inferolaterally from the C7 spinous process towards the second rib angle (Figure 9A), lateral to which the dorsal scapular artery might be seen. The other three digitations of the serratus posterior superior can be seen sequentially deep to the levator scapulae or rhomboids while moving the transducer caudally (Figure 9B). Protraction of the examined shoulder can expose the rib angle and facilitate visualization of the muscle’s lateral attachment.

**Figure 10:**
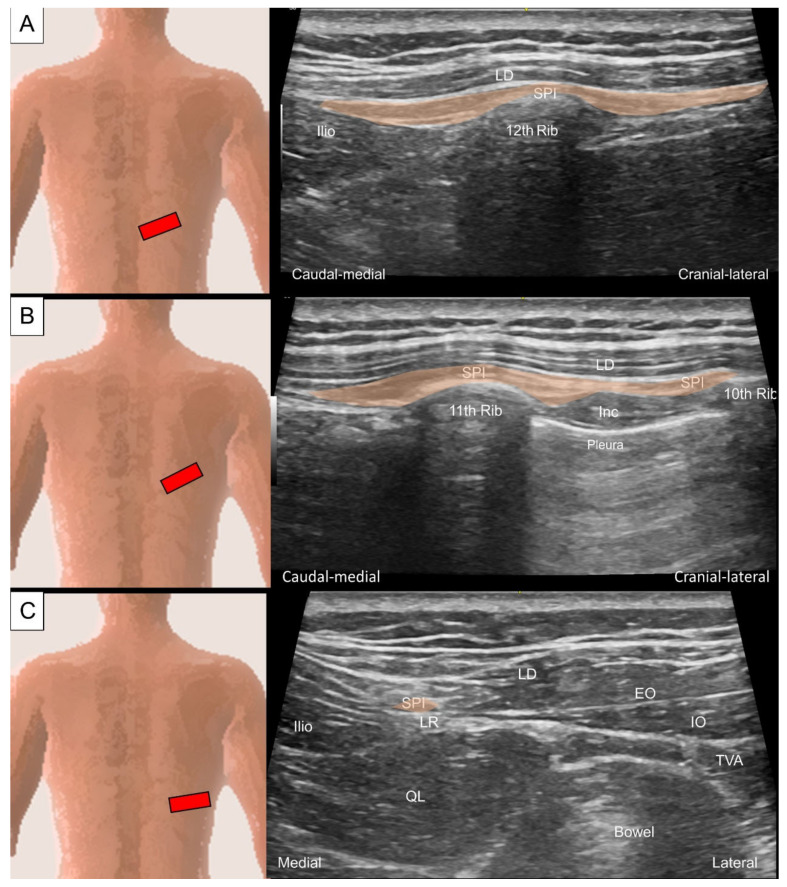
Ultrasound imaging of the serratus posterior inferior (SPI) at the level of (**A**) the 12th rib, (**B**) between the 11th and 10th ribs, and (**C**) beside the lateral raphe (LR) where the quadratus lumborum (QL), paraspinal muscles, and abdominal muscles merge. LD, latissimus dorsi; Ilio, iliocostalis; Inc, intercostal muscle; EO, external oblique muscle; IO, internal oblique muscle; TVA, transversus abdominis.

To scan the serratus posterior inferior, the transducer is first placed on the 12th rib in the center of the screen and the transducer is pivoted 45 degrees (clockwise for the left side and counterclockwise for the right side) to align with the muscle’s long axis. The serratus posterior inferior is seen superficial to the 12th rib and deep to the latissimus dorsi (Figure 10A). This is the “home position” for scanning this muscle. By sliding the transducer more cranially, the serratus posterior inferior can be traced to the more proximal attachments on the 11th to ninth ribs (Figure 10B). Then, the transducer is slid back to the home position and then more caudally to the muscle’s attachment on the spinous processes of the thoracolumbar vertebrae. Here, the serratus posterior inferior, together with the latissimus dorsi, forms the posterior layer of thoracolumbar fascia. A transitional area where the serratus posterior inferior attaches to the cranial end of the lateral raphe (a dense connective tissue complex where the abdominal myofascial structures join the paraspinal muscle sheath at the lateral border of the paraspinal muscles) can be visualized (Figure 10C) [20].

#### 3.1.3. Clinical Relevance

Myofascial pain syndromes caused by trigger points in the serratus posterior superior or inferior are important differential diagnoses for interscapular or thoracolumbar region pain [6]. Ultrasound-guided injection of the serratus posterior superior muscle has been proposed as a safe and effective method to aid in the diagnosis and treatment of the aforementioned pain condition [21,22].

## 4. Intrinsic Muscles of the Cervical Region

### 4.1. Splenius Capitis and Longissimus Capitis

#### 4.1.1. Anatomy

**Figure 11:**
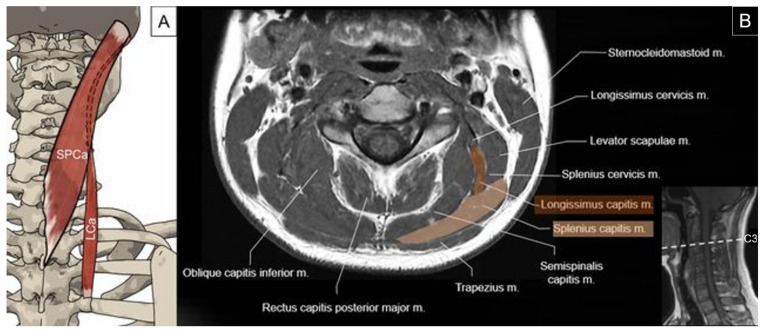
Illustration (**A**) and axial view of magnetic resonance imaging (**B**) for the splenius capitis (SPCa) and longissimus capitis (LCa) muscles.

The superficial layer of the intrinsic back muscle is composed of the splenius capitis and cervicis, which are located in the lateral and posterior neck region. The splenius capitis originates from the spinous processes of the C7 to T3 (or T4) vertebrae and supraspinous ligaments and inserts on the mastoid process of the temporal bone and the occipital bone just below the lateral third of the superior nuchal line. The splenius captis lies deep to the trapezius and is superficial to the semispinalis capitis and longissimus capitis. The upper part of the muscle lies beneath the sternocleidomastoid muscle. Unilateral contraction of the splenius capitis can rotate the head ipsilaterally, while bilateral contraction can extend the head [2,23] (Figure 11).

The longissimus muscle belongs to the intermediate column of the erector spinae group and comprises the longissimus capitis, longissimus cervicis, and longissimus thoracis. The longissimus capitis originates from the transverse processes of the lower three or four cervical and upper four thoracic vertebrae. It travels with an oblique upward, forward, and outward orientation across the lateral edge of the semispinalis capitis and inserts on the posterior margin of the mastoid process under the cover of the splenius capitis and sternocleidomastoid muscles [2,23]. It has an intramuscular tendon near the insertion on the mastoid process [24]. Unilateral contraction of the longissimus capitis can flex the head to the same side, while bilateral contraction can extend the head (Figure 11).

#### 4.1.2. Sonographic Scanning

**Figure 12:**
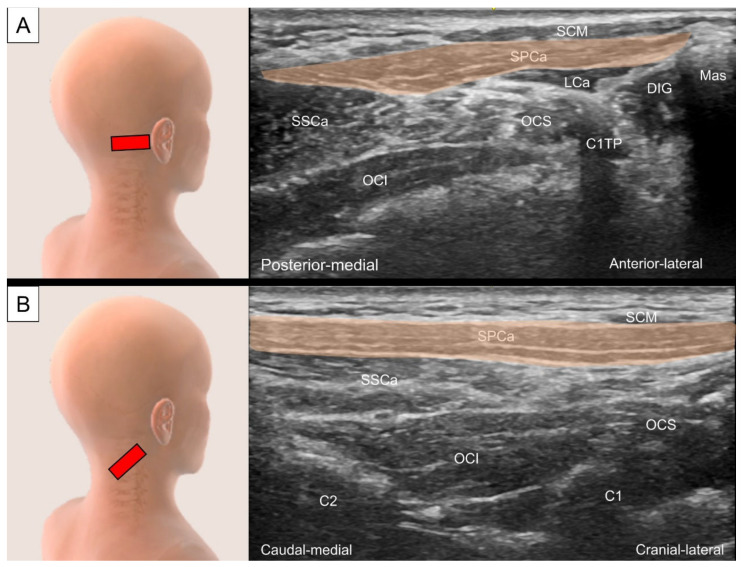
Ultrasound imaging of the splenius capitis (SPCa, skin color shade) in the (**A**) short and (**B**) long axis. SCM, sternocleidomastoid muscle; Mas, mastoid process; DIG, digastric muscle; LCa, longissimus capitis; OCS, obliquus capitis superior; SSCa, semispinalis capitis; OCI, obliquus capitis inferior; C1TP, transverse process of the first cervical vertebrae.

To locate the splenius capitis, place the transducer transversely just posterior to the tip of the mastoid process. The sternocleidomastoid, splenius capitis, and longissimus capitis can be visualized as three muscle layers from superficial to deep (Figure 12A). To view the muscle’s long axis, the transducer is adjusted to align with the line connecting the mastoid process and C7 spinous process (Figure 12B).

**Figure 13:**
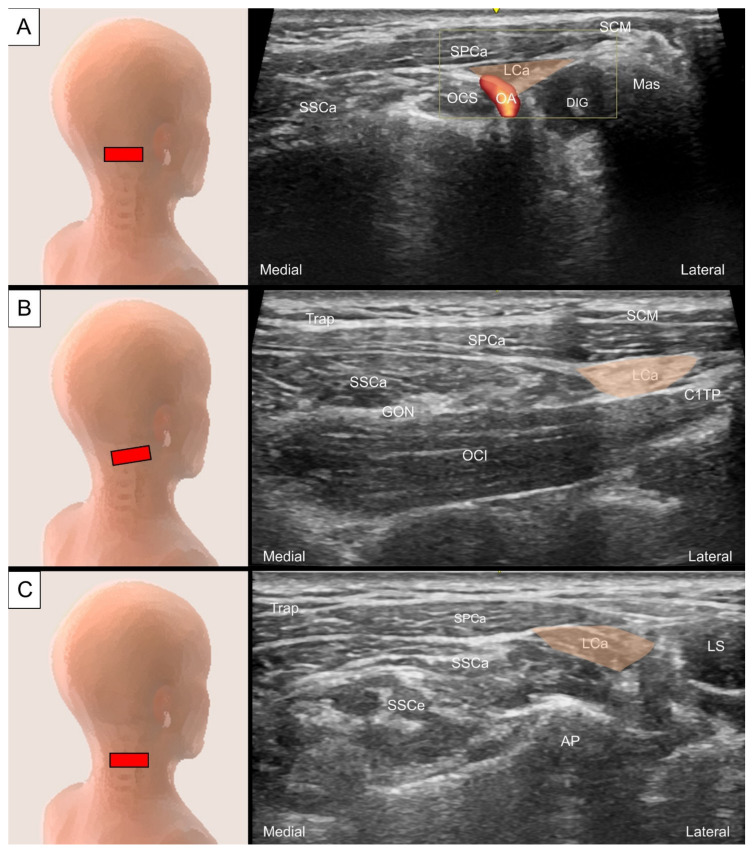
Ultrasound imaging of the longissimus capitis (LCa) at the level of the (**A**) mastoid process, (**B**) transverse process of the first cervical vertebrae and (**C**) middle cervical region. SCM, sternocleidomastoid muscle; SPCa, splenius capitis; Mas, mastoid process; DIG, digastric muscle; OA, occipital artery; SSCa, semispinalis capitis; OCS, obliquus capitis superior; OCI, obliquus capitis inferior; C1TP, transverse process of the first cervical vertebrae; GON, greater occipital nerve; SSCe, semispinalis cervicis; LS, levator scapulae; AP, articular pillar.

The occipital artery can be observed coursing either medially (deep) or laterally (superficial) to the longissimus capitis (Figure 13A) [25]. Tracing caudally, an intramuscular tendon can be seen at the level between the C2 vertebra and the mastoid process (Figure 13B). The longissimus capitis is then observed anterior to the lateral edge of the semispinalis capitis and posterior to the levator scapulae (Figure 13C).

#### 4.1.3. Clinical Relevance

The splenius capitis and longissimus capitis are important targets of botulinum toxin injection for treating cervical dystonia [24]. Since the occipital artery may course around the longissimus muscle, injection under ultrasound guidance could help avoid inadvertent injury to the artery.

### 4.2. Iliocostalis/Longissimus/Splenius Cervicis

#### 4.2.1. Anatomy

The iliocostalis is the most lateral column of the erector spinae group. It comprises the iliocostalis cervicis, iliocostalis thoracis, and iliocostalis lumborum. This muscle group functions to laterally flex the vertebral column to the ipsilateral side while contracting unilaterally, and to extend the vertebral column while contracting bilaterally. The iliocostalis cervicis originates from the angle of the third to sixth ribs and inserts into the posterior tubercle of transverse processes of C4 to C6 [2,23].

The longissimus cervicis muscle originates from the summits of the transverse processes of the upper four or five thoracic vertebrae and ascends to insert on the posterior tubercles of the transverse processes of C2 to C6 vertebrae [2,23]. The function of the longissimus cervicis muscle is to laterally flex the neck while contracting unilaterally and to extend the neck while contracting bilaterally.

**Figure 14:**
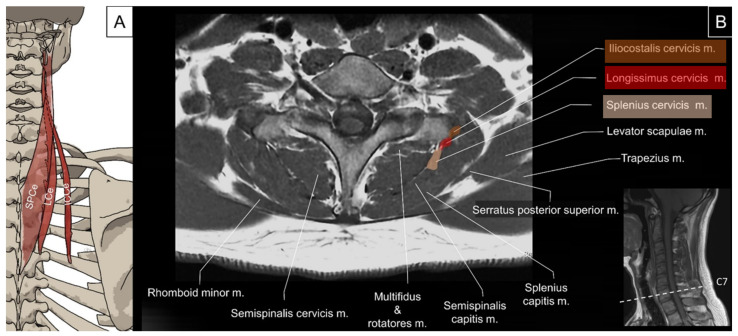
Illustration (**A**) and axial view of magnetic resonance imaging (**B**) for the splenius cervicis (SPCe), longissimus cervicis (LCe) and iliocostalis cervicis (ICCe).

The splenius cervicis muscle originates from the spinous processes of T3 to T6 and inserts into the transverse processes of C1–C3. It may merge with the splenius capitis but extends more caudally [23]. Together with the longissimus capitis and longissimus cervicis muscles, the splenius cervicis muscle constitutes a common lateral muscle mass in the lateral cervical region [24]. The splenius cervicis muscle is deep to the serratus posterior superior, rhomboids, levator scapulae, and trapezius muscles and superficial to the transversospinalis muscles. The function of the splenius cervicis muscle is to ipsilaterally rotate the upper cervical spine while contracting unilaterally and to extend the upper cervical spine while contracting bilaterally (Figure 14).

#### 4.2.2. Sonographic Scanning

**Figure 15:**
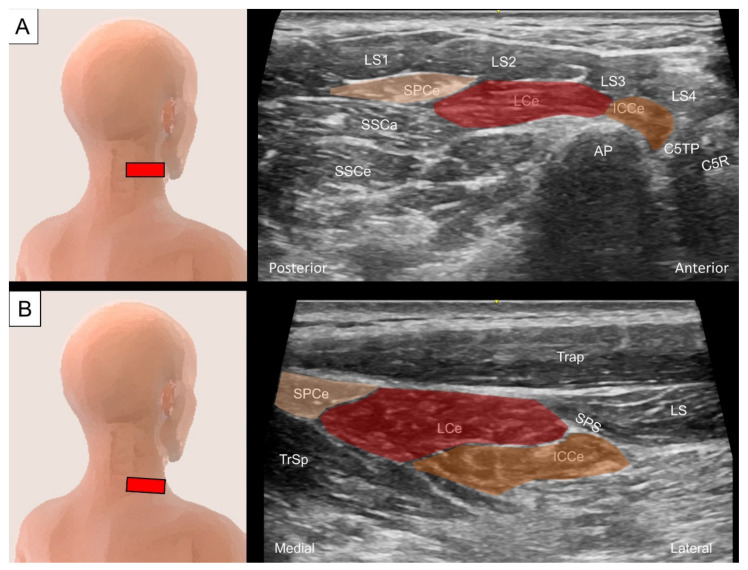
Ultrasound imaging of the splenius cervicis (SPCe, skin color shade), longissimus cervicis (LCe, red shade), and iliocostalis cervicis (ICCe, brown shade) at the (**A**) middle and (**B**) lower cervical level. LS, levator scapulae (1, 2, 3 and 4 indicate different bundles of the levator scapulae); SSCa, semispinalis capitis; SSCe, semispinalis cervicis; AP, articular pillar; C5TP; transverse process of the fifth cervical vertebrae; C5R, fifth cervical root; Trap, trapezius; SPS, serratus posterior superior; TrSp, transversospinalis.

The differentiation of the iliocostalis cervicis, longissimus cervicis, and splenius cervicis is difficult under ultrasound scanning as they overlap with their insertion on the posterior tubercle of the cervical transverse processes. Deep to the four bellies of the levator scapulae, the aforementioned muscles are identified superficially to the cervical articular column and laterally to the semispinalis capitis and longissimus capitis (Figure 15A). At this level, the iliocostalis cervicis, longissimus cervicis, and splenius cervicis are arranged from lateral to medial as they attach to the rib angles, thoracic transverse processes, and spinous processes, respectively. By sliding the transducer caudally towards the cervical–thoracic junction, the serratus posterior superior can be seen at the lateral border of the aforementioned muscle group (Figure 15B).

#### 4.2.3. Clinical Relevance

The three muscles are relatively smaller than other paraspinal muscle. In this sense, they are rarely the main generator of back pain.

### 4.3. Transversospinalis (Semispinalis Capitis/Semispinalis Cervicis/Multifidus)

#### 4.3.1. Anatomy

**Figure 16:**
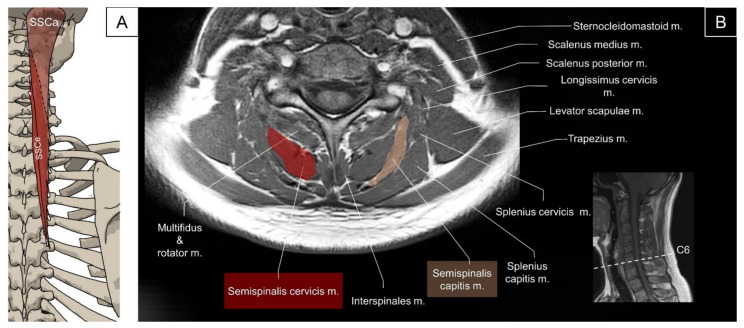
Illustration (**A**) and axial view of magnetic resonance imaging (**B**) for the semispinalis capitis (SSCa) and semispinalis cervicis (SSCe).

The semispinalis muscle group comprises the semispinalis capitis, semispinalis cervicis, and semispinalis thoracis. The semispinalis capitis originates from the superior articular processes of the lower four cervical vertebrae and the transverse processes of the upper six or seven thoracic vertebrae, and inserts on the zone between the superior and inferior nuchal lines of the occipital bone. It lies deep to the trapezius and splenius and is medial to the longissimus capitis and cervicis. The semispinalis capitis has two heads, the medial and the lateral heads, which are separated by a thick tendinous septum at the proximal part [2]. Both the great occipital and third occipital nerves penetrate the medial head of the semispinalis capitis [26]. The function of the semispinalis capitis is to ipsilaterally flex the head while contracting unilaterally and to extend the head while contracting bilaterally. The semispinalis cervicis originates from the posterior surfaces of the transverse processes of the upper five or six thoracic vertebrae and inserts on the spinous processes of the C2 to C5 vertebrae. The main function of the semispinalis cervicis is to extend the neck (Figure 16) [2].

The multifidus in the cervical region originates from the superior articular processes of C4 to C7 vertebrae and attaches to the spinous processes two to four segments above the origin. It covers the lamina of the underlying vertebrae. The main function of this muscle is to extend the cervical vertebrae [2]. The schematic illustration would not be provided in this session as the muscles have been mostly covered by other sessions.

#### 4.3.2. Sonographic Scanning

**Figure 17:**
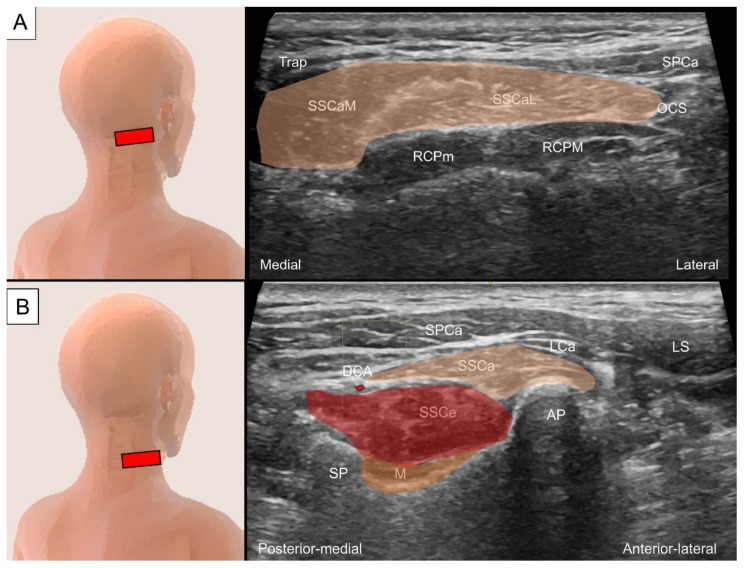
Ultrasound imaging of the transversospinalis at (**A**) between the C1 and C2 and (**B**) middle cervical level. SSCa, semispinalis capitis (skin color shade; M, medial bundle; L, lateral bundle); SSCe, semispinalis cervicis (red color shade); M, multifidus (brown color shade); SPCa, splenius capitis; LCa, longissimus capitis; Trap, trapezius; RCPM, rectus capitis posterior major; RCPm, rectus capitis posterior minor; OCS, obliquus capitis superior; LS, levator scapulae; DCA, deep cervical artery; AP, articular pillar; SP, spinous process.

To locate the semispinalis capitis, the transducer is placed transversely at the para-median suboccipital level. The semispinalis capitis is visualized in its short axis deep to the trapezius and medial to the splenius capitis. At the C1 vertebral level, an intra-muscular tendinous septum is seen, dividing the muscle into the medial and lateral heads (Figure 17A). Continuing to slide the transducer caudally to the C2 level, the greater occipital nerve can be seen as a hyperechoic oval structure lying between the semispinalis capitis and the underlying obliquus capitis inferior muscle. Below the C2 level, the muscle fibers of the semispinalis capitis can be seen coursing laterally and caudally to attach to the superior articular pillars of the lower cervical spine (Figure 17B). To scan the semispinalis cervicis, the transducer is placed transversely adjacent to the C2 spinous process and slid caudally to see the muscle emerging from the spinous process. The deep cervical artery could serve as a landmark, which lies in the fascial plane between the semispinalis capitis and cervicis (Figure 17B) [27]. The multifidus is located deep to the semispinalis cervicis muscle, lateral to the junction of the spinous process and the lamina, dorsal to the lamina of the vertebrae, and medial to the articular pillar (Figure 17B) [28].

#### 4.3.3. Clinical Relevance

The medial head of the semispinalis capitis muscle at the C1 level serves as a suitable target for injecting the greater and third occipital nerves under one needle entry [26]. Semispinalis capitis muscle atrophy has been reported in patients with chronic non-specific neck pain [29]. Furthermore, irritation of the semispinalis cervicis and cervical multifidus would refer to the occipital, suboccipital, neck, and superior medial scapular region [6].

## 5. Intrinsic Muscles of the Thoracic and Lumbar Regions

### 5.1. Iliocostalis Thoracis, Longissimus Thoracis, Semispinalis Thoracis, and Thoracic Multifidus

#### 5.1.1. Anatomy

The iliocostalis thoracis originates from the angles of the lower six ribs and inserts on the transverse process of the seventh cervical vertebra and the angles of the upper six ribs. It lies laterally to the iliocostalis cervicis and medially to the attachment of the serratus posterior superior.

The longissimus thoracis is the largest component of the erector spinae, consisting of thoracic and lumbar parts. The thoracic part of the longissimus thoracis has a short rostral tendon and a long caudal tendon. The caudal tendon forms a wide aponeurosis that attaches to the lumbar spinous processes, supraspinous ligament, median sacral crest, posterior surface of the sacrum, and the dorsal segment of the iliac crest. The rostral tendon inserts on the first four thoracic transverse processes, as well as the lower eight thoracic transverse processes and the adjacent ribs [2,23].

**Figure 18:**
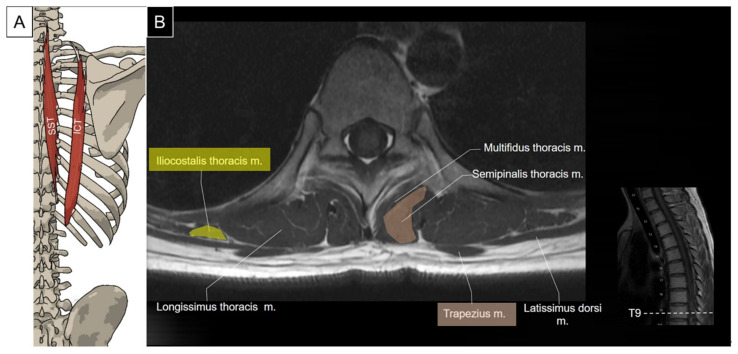
Illustration (**A**) and axial view of magnetic resonance imaging (**B**) for the semispinalis thoracis (SST) and iliocostalis thoracis (ICT) muscles. The longissimus thoracis and thoracic multifidus would be illustrated in the latter session.

The semispinalis thoracis originates from the transverse processes of the sixth to tenth thoracic vertebrae and passes medially and cranially to insert on the spinous processes of the lower two cervical and the upper four thoracic vertebrae. This muscle lies deep to the spinalis thoracis and superficial to the multifidus. Its primary function is to extend the thoracic spine (Figure 18) [2].

The thoracic multifidus originates from the transverse processes of the thoracic vertebrae near their base. The fibers take a craniomedial course to insert on the spinous processes at two to five levels above their origin [2].

#### 5.1.2. Sonographic Scanning

**Figure 19:**
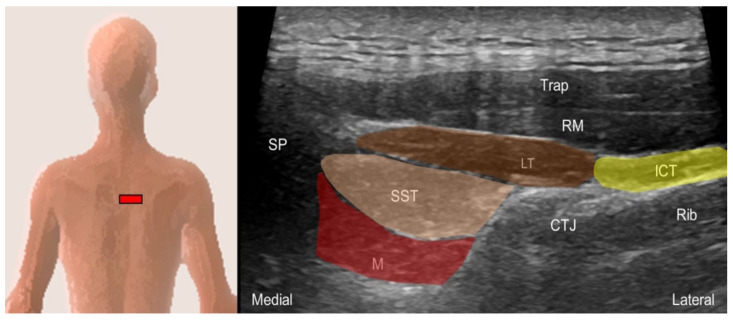
Ultrasound imaging of the iliocostalis thoracis (ICT, yellow shade), longissimus thoracis (LT, brown shade), semispinalis thoracis (SST, skin color shade) and thoracic multifidus (M, red shade) at the thoracic level. Trap, trapezius; RM, rhomboid major; CTJ, costotransverse joint; SP, spinous process.

The transducer is placed transversely at the interscapular region near the examined side. The iliocostalis thoracis and longissimus thoracis muscles are visualized as the two muscle bulks, from lateral to medial, lying deep to the serratus posterior superior or rhomboids. The longissimus thoracis muscle lies on the thoracic transverse processes, appearing as the largest muscle among the erector spinae, while the iliocostalis thoracis muscle lies lateral to it. Moving the shoulder forward can facilitate the visualization of these three muscles by abducing the scapulae. The semispinalis thoracis and thoracic multifidus muscles are located between the spinous process and the transverse process. The multifidus muscle lies deeper, just lateral to the junction of the spinous process and the lamina (Figure 19).

#### 5.1.3. Clinical Relevance

The trigger points of the thoracic intrinsic back muscles can lead to myofascial pain syndrome over the back region with cephalad/caudal radiations [6]. Ultrasound can be used to detect muscle edema and the loss of normal muscle echotexture in patients with thoracic intrinsic back muscle injuries, which can aid in planning a specific rehabilitation program [30].

### 5.2. Iliocostalis Lumborum, Longissimus Thoracis, and Lumbar Multifidus

#### 5.2.1. Anatomy

The lumbar part of the longissimus thoracis originates from the lumbar intermuscular aponeurosis, attaching to the medial ilium and dorsal sacroiliac ligament. Lateral to the lumbar part of longissimus thoracis is the iliocostalis lumborum. Moreover, the lumbar part of longissimus thoracis is separated from the multifidus by a wide cleavage plane filled with fat and veins [2]. The fascicles pass slightly medially and cranially to insert on the accessory process and transverse process of the lumbar vertebrae.

**Figure 20:**
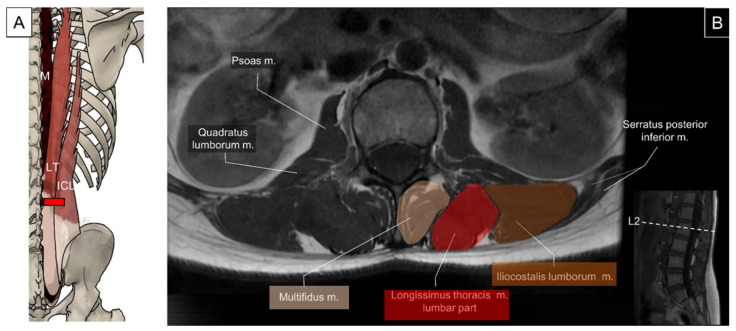
Illustration (**A**) and axial view of magnetic resonance imaging (**B**) for the iliocostalis lumborum (ICL), longissimus thoracis (LT), and lumbar multifidus (M).

The lumbar multifidus originates from the mammillary processes of the lumbar vertebrae and the posterior surface of the sacrum. It courses medially and cranially to insert on the spinous processes, two to five levels above their origin (Figure 20) [2,31].

#### 5.2.2. Sonographic Scanning

**Figure 21:**
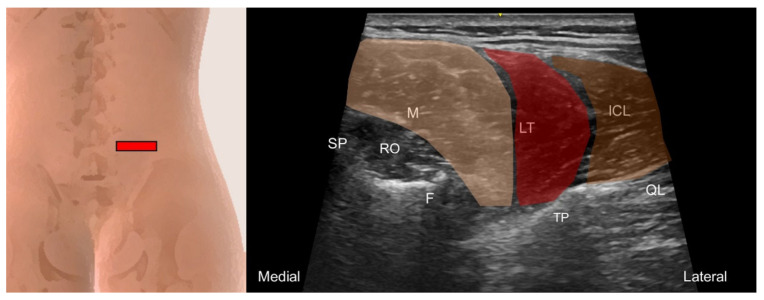
Ultrasound imaging of the iliocostalis lumborum (ICL, brown shade), longissimus thoracis (LT, red shade), and multifidus (M, skin color shade) at the lumbar region. SP, spinous process, RO, rotatores (can be categorized as part of the multifidus that connects the mammillary process and the spinous process at the same level or one level above); F, facet; TP, transverse process; QL, quadratus lumborum.

According to findings from Romero et al.’s research, ultrasound imaging emerges as a dependable means for evaluating lumbar multifidus muscle thickness in dynamic positions, encompassing individuals with and without non-specific chronic low back pain [32]. By placing the transducer transversely on the upper lumbar spine adjacent to the spinous process, the iliocostalis lumborum, longissimus thoracis, and multifidus can be sequentially visualized from lateral to medial. The multifidus is confined to the region over the lamina at the upper lumbar levels, and it progressively expands to cover the sacral bone when the transducer is slid caudally from the L3 level. Lateral to the multifidus, the longissimus thoracis and iliocostalis lumborum converge onto the transverse process (Figure 21).

#### 5.2.3. Clinical Relevance

The trigger points in the lumbar intrinsic back muscles can cause myofascial pain syndrome over the lumbar and buttock region [6]. Among the lumbar paraspinal muscles, the multifidus is the most crucial muscle as it contributes to almost two thirds of spinal stability and is predominantly atrophied in patients with chronic low back pain [33].

## 6. Other Associated Muscles

### 6.1. Sub-Occipital Muscles

#### 6.1.1. Anatomy

**Figure 22:**
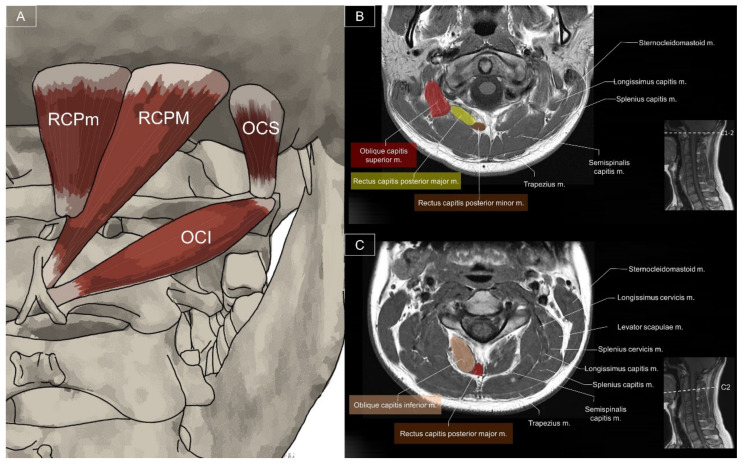
Illustration (**A**) and axial view of magnetic resonance imaging for the sub-occipital muscle at the C1-2 (**B**) and C2 levels (**C**). OCS, obliquus capitis superior; RCPm, rectus capitis posterior minor; RCPM, rectus capitis posterior major; OCI, obliquus capitis inferior.

The suboccipital muscles are a group of four muscles located inferior to the occipital bone, including the obliquus capitis inferior (OCI), obliquus capitis superior (OCS), rectus capitis posterior major (RCPM), and rectus capitis posterior minor (RCPm). The OCI originates from the C2 spinous process and passes laterally and cranially to insert onto the C1 transverse process, acting to ipsilaterally rotate the head. The greater occipital nerve courses on top of the OCI [34]. The OCS originates from the C1 transverse process and inserts onto the lateral half of the inferior nuchal line on the occipital bone, acting to extend and bend the head laterally [34]. The RCPM originates from the C2 spinous process and inserts on the inferior nuchal line of the occipital bone, functioning to extend the neck and ipsilaterally rotate the head [34]. The RCPm originates from the C1 posterior tubercle and inserts on the inferior nuchal line. Like the RCPM, the RCPm functions to extend the neck and ipsilaterally rotate the head (Figure 22) [34].

#### 6.1.2. Sonographic Scanning

**Figure 23:**
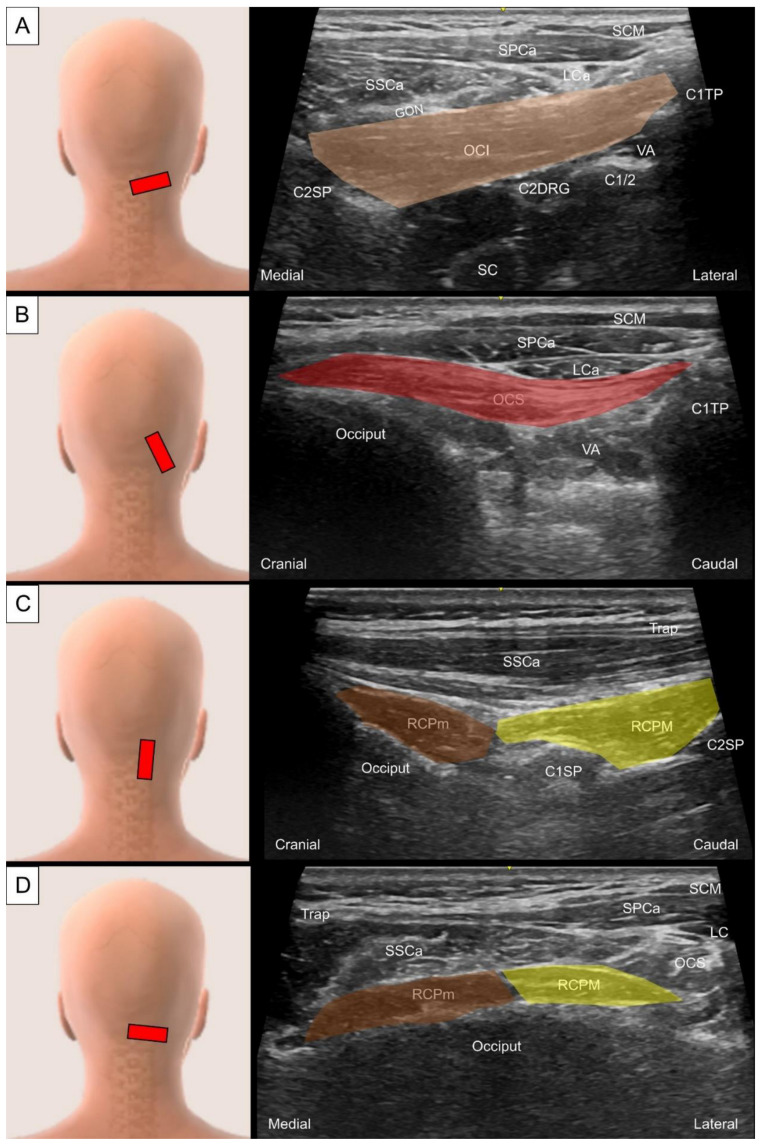
Ultrasound imaging of (**A**) the obliquus capitis inferior (OCI, skin color shade) above the C2 lamina, (**B**) the obliquus capitis superior (OCS, red shade) in its long axis, and rectus capitis posterior minor (RCPm, brown shade) and rectus capitis posterior major (RCPM, yellow shade) in the (**C**) oblique- and (**D**) short-axis view. SCM, sternocleidomastoid muscle; SPCa, splenius capitis; SSCa, semispinalis capitis; LC, longissimus capitis; C1TP, transverse process of the first cervical vertebrae; VA, vertebral artery; C2DRG, second cervical dorsal root ganglion; C2SP, spinous process of the second cervical vertebrae; C1SP, spinous process of the first cervical vertebrae; SC, spinal cord; Trap, trapezius; GON, greater occipital nerve.

To identify the OCI, the examiner should place the medial end of the transducer at the C2 spinous process and pivot the lateral end towards the C1 transverse process. The OCI can be seen in its long axis above the C2 lamina (Figure 23A). The greater occipital nerve can be visualized as a small hyperechoic oval structure lying between the OCI and the semispinalis capitis. Deep to the OCI muscle, the C1/2 facet can be seen with the vertebral artery on its lateral aspect and the C2 dorsal root ganglion on its medial aspect. For scanning of the OCS, the examiner should keep the lateral end of the transducer on the C1 transverse process and pivot the medial end towards the occiput. The OCS muscle can then be seen in its long axis, with tendinous attachment on the C1 transverse process and muscular attachment on the occiput (Figure 23B). To scan the RCPm and RCPM, the transducer should be placed in a paramedian sagittal oblique plane at the C1 and C2 level, allowing for visualization of the two muscles at the attachment on the spinous processes in the oblique axis view (Figure 23C). By placing the transducer in the horizontal plane between the occiput and C1 spinous process, the OCS, RCPM, and RCPm in their short axis can be clearly demonstrated (Figure 23D).

#### 6.1.3. Clinical Relevance

The suboccipital muscles are vulnerable to rapid deceleration (whiplash) injuries, which can result in spasm, strain, or tear of the suboccipital muscles or damage to the suboccipital nerves. Myofascial pain syndrome caused by trigger points in the suboccipital muscles can cause pain radiating from the occiput to the temporal region, eyes, and forehead [6,34].

### 6.2. Serratus Anterior

#### 6.2.1. Anatomy

**Figure 24:**
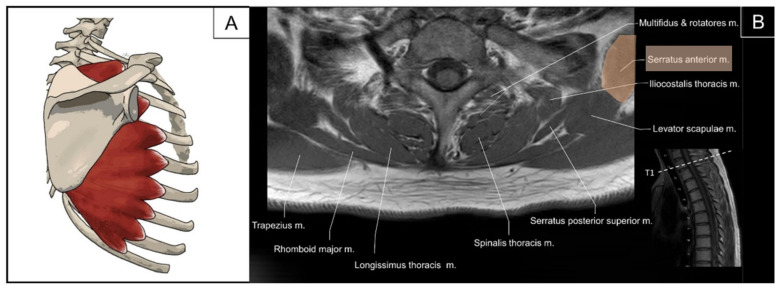
Illustration (**A**) and axial view of magnetic resonance imaging (**B**) for the serratus anterior muscle.

The serratus anterior originates from the first to eighth or ninth ribs and inserts on the medial border of the scapula [2,35]. The fibers of the serratus anterior pull the scapula forward around the chest wall, allowing anteversion and protraction of the arm. Furthermore, the serratus anterior acts in conjunction with the upper and lower fibers of the trapezius muscle to rotate the scapula upwards, allowing for overhead lifting. When the shoulder girdle is fixed, all the serratus anterior works together to lift the ribs, assisting respiration (Figure 24).

#### 6.2.2. Sonographic Scanning

**Figure 25:**
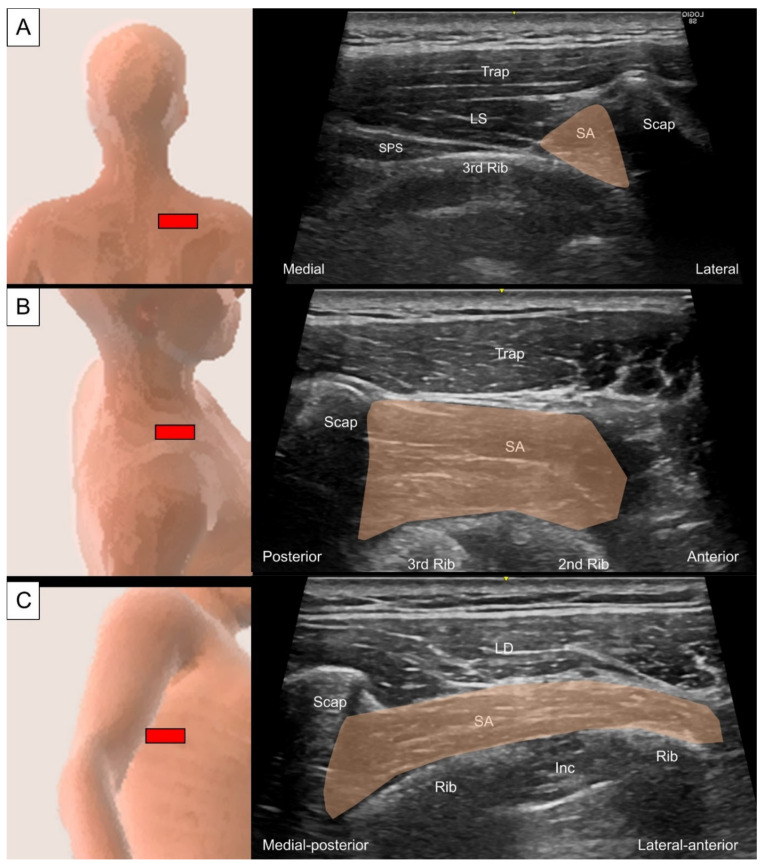
Ultrasound imaging of the serratus anterior (SA, skin color shade) (**A**) lying underneath the scapula, (**B**) crossing the intercostal spaces to insert on the ribs, and (**C**) running at the lateral trunk region. Trap, trapezius; Scap, scapula; LS, levator scapulae; SPS, serratus posterior superior; LD, latissimus dorsi; Inc, intercostal muscle.

Although the upper fibers of the serratus anterior lie deep to the scapula, which blocks the ultrasound beam for visualizing the muscle, it can still be accessed at the superior medial border of the scapula. Firstly, the transducer is placed horizontally at the level of the scapular spine with the subject in a prone position. The upper fibers of the serratus anterior can be seen in the short axis as a triangular-shaped hyperechoic structure underneath the scapula (Figure 25A). Herein, the serratus anterior should be distinguished from the levator scapulae, which also attaches to the superior medial border of the scapula. By pivoting the transducer to the sagittal plane, the muscle can be further confirmed by seeing it crossing the intercostal spaces to insert on the ribs (Figure 25B) [12]. To examine the lower fibers, the subject assumes a decubitus position with the examined side on top. The transducer is placed transversely at the inferior angle of the scapula towards the lower ribs to scan the individual lower digitations of this muscle (Figure 25C).

#### 6.2.3. Clinical Relevance

Patients with trigger points over the serratus anterior complain of pain localized along the midaxillary line, from the fifth to seventh ribs. Ultrasound-guided injection of the serratus anterior is an effective procedure to relieve pain and decrease the risk of pneumothorax in comparison with the palpation-guided procedure [35,36].

### 6.3. Quadratus Lumborum and Psoas Major

#### 6.3.1. Anatomy

**Figure 26:**
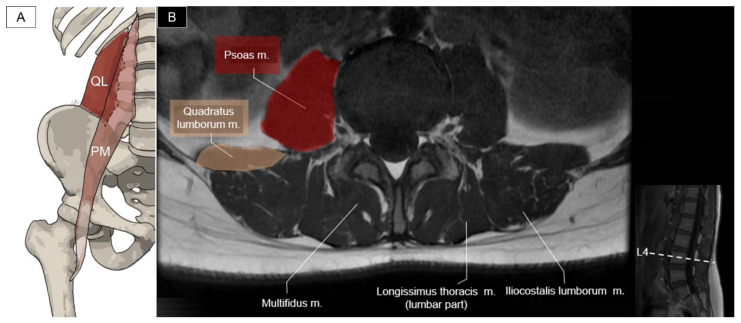
Illustration (**A**) and axial view of magnetic resonance imaging (**B**) for the quadratus lumborum (QL) and psoas major (PM) muscle.

The quadratus lumborum originates from the iliac crest and the iliolumbar ligament and inserts on the twelfth rib and the transverse processes of the L1 to L4 vertebrae. The function of the quadratus lumborum is to laterally flex the lumbar spine, elevate the pelvis, or extend the lumbar spine (if bilaterally contracted) [37]. The psoas major originates from the vertebral bodies, the intervertebral discs (around T12 to L4), and the transverse processes (L1 to L5). It joins the iliacus muscle distally to form the iliopsoas and inserts onto the lesser trochanter of the femur. This muscle is a major contributor to flexion of the hip joint. Unilateral contraction of the psoas helps the lateral rotation of the thigh and bilateral contraction can raise the trunk from the supine position (Figure 26) [38].

#### 6.3.2. Sonographic Scanning

**Figure 27:**
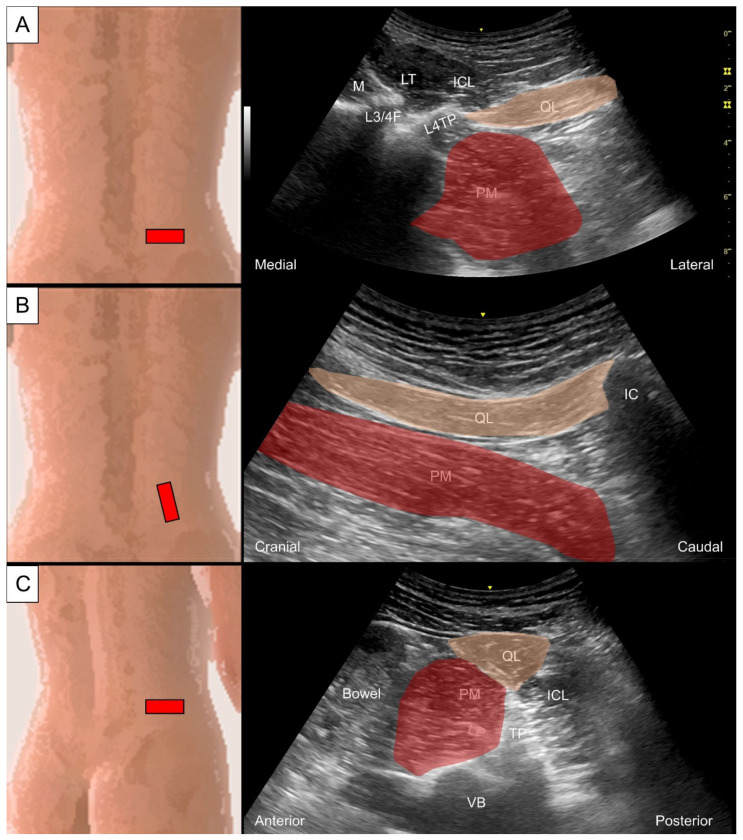
Ultrasound imaging for the quadratus lumborum (QL, skin color shade) and psoas major (PM) in their (**A**) short and (**B**) long axes; the shamrock sign made of the aforementioned muscles and iliocostalis lumborum (ICL) (**C**). M, multifidus; LT, longissimus thoracis; IC, iliac crest; L3/4F, facet joint between the L3 and L4 vertebrae; L4TP, transverse process of the L4 vertebrae; TP, transverse process.

Two approaches could be utilized for the scanning of the quadratus lumborum and psoas major. In the first approach, the subject assumes a prone posture and the transducer is placed adjacent to the midline of the lumbar spine on the examined side and toggled upwards and downwards to locate the transverse processes. The short axis of the quadratus lumborum is seen deep to the paraspinal muscles (iliocostalis, longissimus, and multifidus), attaching onto the transverse processes (Figure 27A). Changing the transducer to a craniomedial to caudolateral orientation, the long axis of quadratus lumborum is seen to course from the transverse processes to attach on the iliac crest (Figure 27B). For visualizing the psoas major in the short axis, the transducer is tilted inwards, and the psoas major is seen located at the anterolateral aspect of the lumbar vertebrae. In the second approach, the subject assumes a decubitus position with the examined side on top and the transducer is placed transversely on the side of the lumbar spine. The transducer is toggled upwards and downwards for scanning the transverse process. The quadratus lumborum appears triangular (instead of flat in the first approach), situating at the apex of the transverse process, also known as the “shamrock sign” (Figure 27C) [39].

#### 6.3.3. Clinical Relevance

Myofascial pain syndrome caused by the trigger points of the quadratus lumborum is a frequent cause of chronic low back pain. The pain is located over the sacroiliac joint, buttock, greater trochanter, or groin. Trigger points of the psoas major can lead to low back pain with ipsilaterally radiated pain along the lumbar spine or to the groin and knee [6,38]. Ultrasound-guided infiltration is shown to be effective for treating pain over the quadratus lumborum or psoas major [40,41].

## 7. Conclusions

Ultrasound imaging serves as a valuable tool enabling the differentiation of individual paraspinal muscles. This capability enhances the precision of interventions aimed at addressing myofascial pain syndrome. To accurately identify the specific muscle being scanned, it is crucial to have a solid understanding of its origin and insertion. Furthermore, dynamic flexion, extension, and lateral bending of the neck and trunk can be useful in verifying pain generators during body movement. It is worth noting that paraspinal muscles are in close proximity to several vital structures, such as the spinal cord, vertebral artery, and pleura, which necessitates the safe and careful guidance of injections using ultrasound technology. Future studies can be conducted to evaluate whether the echotexture, Doppler activities, and elasticity on ultrasound imaging change after the guided intervention for the affected paraspinal muscles.

## Data Availability

Data are contained within the main text of the manuscript.
